# Unusual Case Report of Thrombotic Microangiopathy of the Small Bowel Following Liver Transplantation, a Possible Immunosuppressant-Induced Disease with Histological and Ultrastructural Findings

**DOI:** 10.1100/tsw.2009.115

**Published:** 2009-10-02

**Authors:** Domenico Piscitelli, Maria Grazia Fiore, Roberta Rossi, Michelina Casiello, Francesca Sanguedolce

**Affiliations:** Department of Pathology, University Hospital, Piazza Giulio Cesare, 70124 Bari, Italy; Department of Pathology, University Hospital, Viale Pinto, 71100 Foggia, Italy

**Keywords:** liver transplantation, TMA, immunosuppressant

## Abstract

Cyclosporin-A (CsA) and tacrolimus (FK-506) are immunomodulating agents used to prevent rejection in organ transplantation. They are both associated with several side effects, including nephrotoxicity and severe hypertension due to vascular injury, which often appears as a microvascular occlusive disorder (thrombotic microangiopathy, TMA). We report the first case of a microvascular occlusive disorder with the features of TMA in the small bowel of an orthotopic liver transplant (OLT) patient after immunosuppressive therapy with CsA and FK506. The patient presented with severe recurrent abdominal colics and distal subocclusion, requiring aggressive surgical treatment. Histological and ultrastructural analysis of the resected specimen disclosed intestinal TMA. Although rare, such a complication should be considered in the differential diagnosis of abdominal colics in patients undergoing immunosuppressant therapy after OLT.

## INTRODUCTION

Cyclosporine A (CsA) and tacrolimus (FK-506) are potent immunosuppressants used with success in solid organ transplant for induction and long-term maintenance. They share a common mechanism of action through selective inhibition of T-helper lymphocyte signaling. They also exert biological effects on nonlymphoid cells, including endothelial cells, probably by activation of vasoconstrictors, such as endothelin, and by blockage of nitric oxide production[1]. In fact, the use of such drugs in transplant patients can be associated with development of adverse side effects (nephrotoxicity, severe hypertension), due to a common underlying mechanism of drug-induced vascular changes.

Thrombotic microangiopathy (TMA) is a group of microvascular occlusive disorders that encompasses a spectrum of three clinical entities, one of which is a well-known complication of CsA and

FK-506 therapy in renal and extrarenal (liver, heart, lung, bowel) transplant recipients; the kidney (transplanted or native) is typically the primarily affected site[2].

Herein, we describe a case of microvascular occlusive disorder with endothelial giant mitochondria in the small bowel after liver transplantation and subsequent immunosuppressant therapy.

## CASE

A 70-kg, 57-year-old, white male underwent orthotopic liver transplantation (OLT) in January 2004 for HBV- and alcohol-induced cirrhosis.

He was started on CsA for rejection prophylaxis according to the standard immunosuppression schedule, then switched to FK-506 and prednisone after 2 months because of initial signs of acute rejection disclosed by core liver biopsy. The successive postoperative course was uneventful, and the patient was discharged and returned to active life.

Thirteen months after the transplantation, he began to complain of severe recurrent abdominal colics. Lab results revealed elevation of total bilirubin (4.15 mg/dl) with normal liver enzymes and coagulation tests. No signs of renal impairment were present.

Plain abdominal CT showed splenomegaly and mild dilatation of intestinal loops. An ultrasound-guided liver core biopsy was performed, which demonstrated a cholestatic cholangiopathy, with normal hepatic architecture (no portal fibrosis or inflammation). Due to persistence of symptoms, in spite of adequate medical therapy, and occurrence of progressive constipation and some degree of distal subocclusion, he underwent small bowel resection.

At gross examination, the resected segment of small bowel, measuring 67 cm in length, featured luminal narrowing and thick walls wrapped by an edematous and hyperemic serosa. The overlying mucosa appeared slightly edematous and bosselated with wide necrotic areas interspersed.

Light microscopic examination showed a moderate amount of chronic inflammatory cells, a thinned mucosa with remarkable necrotic-ulcerative foci, and a markedly thickened and edematous submucosa (Fig. 1a). The diagnosis of intestinal TMA was made due to the presence of submucosal small arteries with peculiar changes: serious structural disarray and diffusely thickened walls, sometimes containing circular amorphous PAS+ material, along with myointimal hyperplasia and foci of fibrinoid necrosis. In their lumina, scattered occlusive and subocclusive fibrin thrombi with a variable degree of early organization were detectable (Fig. 1b,c).

Ultrastructural analysis showed narrowed lumina, confirmed focal necrosis of smooth muscle cells and endothelial cells, and revealed circular protein subendothelial deposits and myointimal fibrosis. Giant mitochondria were present in both endothelial and smooth muscle cells, corresponding to type B (round, oval, or cigar-shaped; dense matrices; crystalloid structures; lipid inclusion bodies; and dense granules), according to Mihatsch et al.[3] (Fig. 1d).

## DISCUSSION

CsA-induced vascular changes were first reported by Sommer et al. in 1985[4] as a cause of graft loss in a series of renal allograft recipients. Since then, the development of vascular changes associated with CsA in solid-organ transplantation, including OLT, has been described by many authors, resulting from endothelial cell injury due to direct or indirect (i.e., autoimmune-mediated) action of the drug.

As a histopathological entity, TMA occurs in the kidney in the setting of postdiarrheal hemolytic uremic syndrome (HUS)[5]. Recently, it has been reported following therapy with other immunosuppressors used to prevent graft-versus-host disease (GVHD), such as sirolimus and FK-506[6], the underlying mechanism being probably the same.

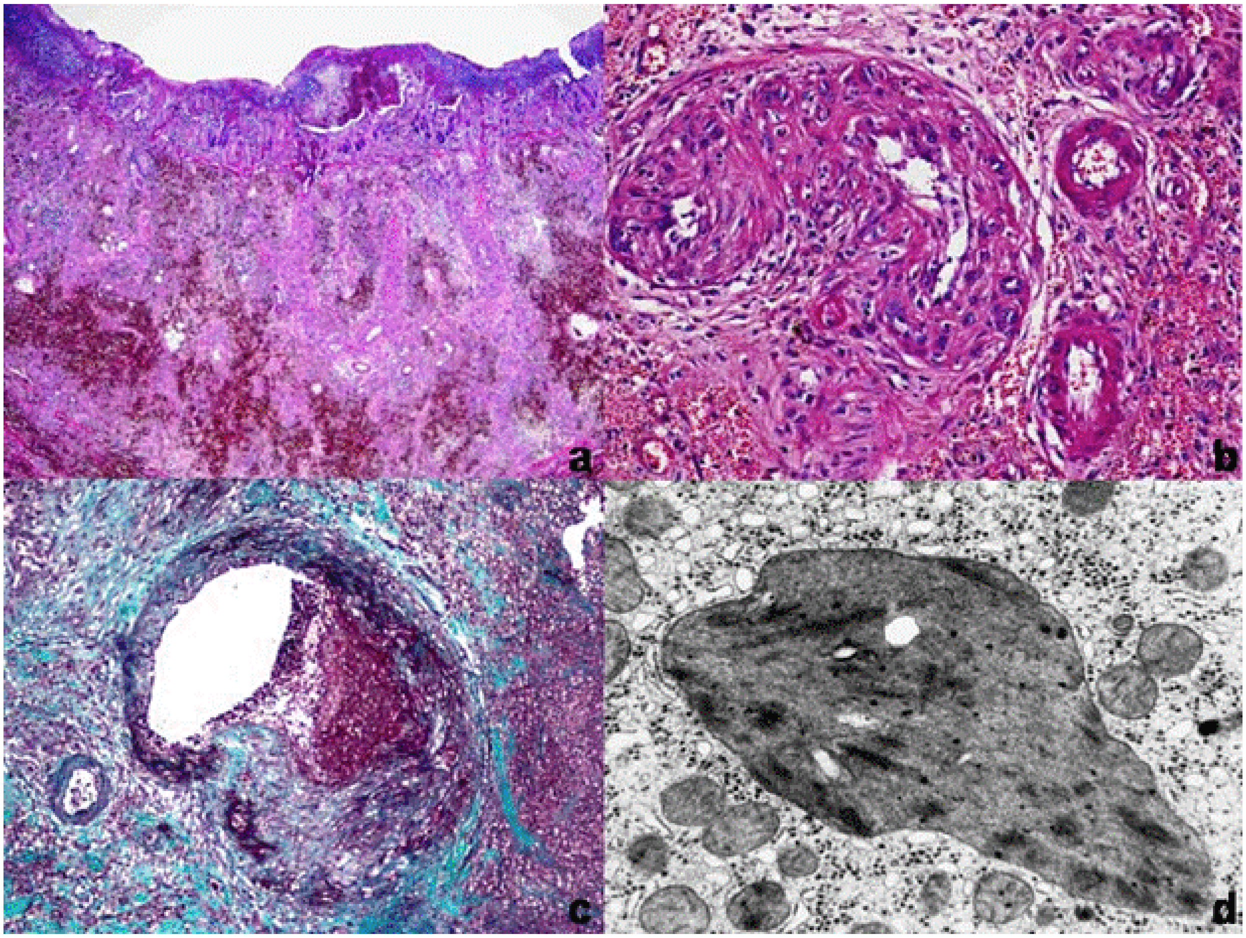

**FIGURE 1.** (a) Severely inflamed intestinal mucosa with necrotic-ulcerative and hemorrhagic areas; (b,c) at higher power, small vessels with structural disarray and diffusely thickened walls, myointimal hyperplasia, circular protein deposits, and fibrin microthrombi, better seen with trichrome stain; (d) type B-giant mitochondria with dense matrices, crystalloid structures, lipid inclusion bodies, and dense granules (see text), electron microscopy, × 15,200.

The localized form of TMA displays a spectrum of lesions of small arterioles and capillaries, ranging from degeneration and fibrosis of vessel walls to occlusion due to fibrin microthrombi, which can ultimately lead to severe ischemic injury, massive tissue necrosis, and organ failure[2,3,7]. The small bowel represents a rare site of TMA, mostly occurring after stem cell transplantation[7]. The diagnosis is made on clinical (history of organ transplantation followed by immunosuppressive therapy, absence of laboratory signs of infection and rejection) and histopathological findings, as described above. Anyway, a major clue is the ultrastructural detection of giant mitochondria featuring organelle swelling, dense matrices, and crystalloid structures[4]; such a combination of changes is strongly suggestive of highly specific subcellular damage due to CsA inhibition of the permeability of the inner mitochondrial membrane, and can be induced in experimental animals treated with CsA[8].

## CONCLUSIONS

In summary, we described here a case of small bowel TMA developing after OLT. The causative drugs were presumed to be CsA and FK-506 on the basis of clinical data, and histological and ultrastructural findings. Although rare, such a complication should be taken into account when a recipient presents with severe abdominal pain, and a proper diagnostic and therapeutic approach should be immediately performed.
